# Reply to “Overstated Claims of Efficacy and Safety. Comment On: Optimal Nutritional Status for a Well-Functioning Immune System Is an Important Factor to Protect against Viral Infections. *Nutrients* 2020, *12*, 1181”

**DOI:** 10.3390/nu12092696

**Published:** 2020-09-03

**Authors:** Philip C. Calder, Anitra C. Carr, Adrian F. Gombart, Manfred Eggersdorfer

**Affiliations:** 1 Faculty of Medicine, University of Southampton and NIHR Southampton Biomedical Research Centre, University Hospital Southampton NHS Foundation Trust, Southampton SO16 6YD, UK; 2 Nutrition in Medicine Research Group, Department of Pathology and Biomedical Science, University of Otago, Christchurch 8140, New Zealand; anitra.carr@otago.ac.nz; 3 Linus Pauling Institute, Department of Biochemistry and Biophysics, Oregon State University, Corvallis, OR 97331, USA; adrian.gombart@oregonstate.edu; 4 Department Internal Medicine, University Medical Center Groningen, 9713 GZ Groningen, The Netherlands; m.eggersdorfer@bluewin.ch

We thank Vorland et al. [1] for their interest in our recent publication [2]. They provide comments in relation to our interpretation of systematic reviews of omega-3 (*n*-3) fatty acids [3] and vitamin C [4,5,6]. The systematic review of *n*-3 fatty acids, sometimes in combination with other nutrients, in acute respiratory distress syndrome (ARDS) was cited in the context of a so-called “cytokine storm” rather than protection from viral, or any other, infection per se. The findings from that systematic review are shown in Table 1.

In our view, these data support our statement that the systematic review identifies “a signiﬁcant improvement in blood oxygenation and signiﬁcant reductions in ventilation requirement, new organ failures, length of stay in the intensive care unit and mortality at 28 days”. In our publication [2] we made a recommendation for intake of eicosapentaenoic acid plus docosahexaenoic acid of 250 mg/day, which is in keeping with many recommendations made by regulatory authorities for these fatty acids [7,8,9] and can be easily achieved through diet (i.e. consumption of fatty fish) [10]. It is important to emphasise that the amount of *n*-3 fatty acids delivered in formulas used in the trials in ARDS is greatly in excess of 250 mg/day, typically several g per day.

Our statements in relation to the systematic review on vitamin C and pneumonia [4] were that there was “a signiﬁcant reduction in the risk of pneumonia with vitamin C supplementation, particularly in individuals with low dietary intakes” and that “in older patients, disease severity and risk of death were reduced with supplementation, particularly in the case where initial plasma levels of vitamin C were low”. Thus, we stress the likely role of vitamin C in those individuals with low intake or status. This is consistent with the findings reported in the systematic review, the abstract of which makes the final conclusion “therapeutic vitamin C supplementation may be reasonable for pneumonia patients who have low vitamin C plasma levels because its cost and risks are low”. In relation to the systematic review on vitamin C and the common cold [5] we stated that “vitamin C supplementation has also been shown to decrease the duration and severity of upper respiratory tract infections, such as the common cold, and signiﬁcantly decrease the risk of infection when given prophylactically in people under enhanced physical stress”. The results section of the abstract of the systematic review states “in adults the duration of colds was reduced by 8% (3% to 12%) and in children by 14% (7% to 21%). In children, 1 to 2 g/day vitamin C shortened colds by 18%. The severity of colds was also reduced by regular vitamin C administration”. Furthermore, the conclusions section of the abstract of the systematic review states “vitamin C may be useful for people exposed to brief periods of severe physical exercise. Regular supplementation trials have shown that vitamin C reduces the duration of colds, but this was not replicated in the few therapeutic trials that have been carried out. Nevertheless, given the consistent effect of vitamin C on the duration and severity of colds in the regular supplementation studies, and the low cost and safety, it may be worthwhile for common cold patients to test on an individual basis whether therapeutic vitamin C is beneficial for them”. We note that risk ratio for the incidence of common colds in those undertaking heavy acute physical activity was 0.48 (95% confidence interval 0.35–0.64). We consider that our statements are consistent with the findings of these two systematic reviews. 

Vorland et al. [1] raise the important question of safety. We do not dispute that safety is of utmost importance, and in accordance with this, we state that “intakes should follow recommended upper safety limits set by expert authorities, such as the European Food Safety Authority and, in the United States, the IOM” and we go on to discuss intakes of vitamins C and D in relation to stated tolerable upper limits and where these vary in children. 

Our review deals with micronutrients (and *n*-3 fatty acids), viral infection and related respiratory disease; it is not restricted solely to COVID-19. There are not yet any published trials of supplementation with individual or mixed micronutrients and either prevention or treatment of COVID-19; we wish to emphasise that we did not give any impression that such evidence currently exists. However, given that there is a substantial body of evidence that micronutrients support the function of many components of the immune system [11] and may reduce risk of incidence or severity of respiratory illness [2,11], we consider that such trials are warranted.

## Figures and Tables

**Table 1:** Summary of the findings of the systematic review and meta-analysis of the effects of n-3 fatty acid rich formulas in patients with acute respiratory distress syndrome [3].

Outcome	Effect	95% CI	*p*
PaO_2_/FiO_2_ at day 4 (Mean difference mmHg)	38.88	10.75–67.02	0.0068
PaO_2_/FiO_2_ at day 8 (Mean difference mmHg)	23.44	1.73–45.15	0.034
Ventilator days (Mean difference d)	−2.24	−3.77–−0.71	0.0042
New organ failure (Relative risk)	0.45	0.32–0.63	<0.00001
Length of intensive care unit stay (Mean difference d)	−3.09	−5.19–−0.99	0.004
28 day mortality (Relative risk)	0.64	0.49–0.84	0.0015
All cause mortality (Relative risk)	0.79	0.59–1.07	Not given

CI, confidence interval.
